# Effects of Dietary Glycerol Monolaurate on Growth Performance, Bile Acid Metabolism, and Intestinal Health in Asian Swamp Eel (*Monopterus albus*)

**DOI:** 10.3390/ani16111575

**Published:** 2026-05-22

**Authors:** Haiyan Liu, Hang Yang, Xiaogang Guo, Menghui Lin, Minjie Zhao, Wenzong Zhou, Haiying Cai

**Affiliations:** 1 College of Biology and Chemical Engineering, Zhejiang University of Science and Technology, Hangzhou 310023, China; 2 Eco-Environmental Protection Research Institute, Shanghai Academy of Agricultural Sciences, Shanghai 201403, China; 3 Key Laboratory of Integrated Rice-Fish Farming Ecosystem, Ministry of Agriculture and Rural Affairs, Shanghai Academy of Agricultural Sciences, Shanghai 201403, China; 4 School of Biosystem Engineering and Food Science, Zhejiang University, Hangzhou 310058, China

**Keywords:** glycerol monolaurate, *Monopterus albus*, growth performance, bile acid, intestinal health

## Abstract

This study investigated the dose-dependent effects of dietary glycerol monolaurate (GML) supplementation on the growth performance, bile acid metabolism, and intestinal health of Asian swamp eel (*Monopterus albus*). A total of 225 healthy eels with an initial body weight of 25.0 ± 2.0 g were randomly divided into three groups and fed a basal diet (control), 0.5 g/kg GML, or 1.0 g/kg GML for 60 days, respectively. The results showed that dietary GML supplementation increased the weight gain rate by 8.75% and decreased the feed conversion ratio by 8.16% in the GML1 group compared with the control. Dietary GML significantly reduced the contents of TP and ALB (*p* < 0.05). Meanwhile, the MDA content in the GML0.5 and GML1 groups decreased significantly by 24.80% and 38.15%, respectively. The contents of total bile acids and secondary bile acids were significantly elevated in the GML1 group (*p* < 0.05). In addition, GML supplementation optimized the intestinal microbial community and significantly reduced the relative abundance of potentially harmful bacteria such as *Nocardia* and *Stenotrophomonas* (*p* < 0.05). In conclusion, under the conditions of this study, dietary GML could effectively the improve growth performance, bile acid metabolism, antioxidant capacity, and intestinal health of *M. albus*. Among the tested concentrations, 1.0 g/kg GML showed the optimal beneficial effects.

## Simple Summary

This study evaluated the effects of dietary glycerol monolaurate (GML) on growth, bile acid metabolism, and intestinal health in Asian swamp eel (*Monopterus albus*). A total of 225 eels were fed diets supplemented with 0, 0.5, or 1.0 g/kg GML for 60 days. The results showed that 1.0 g/kg GML significantly improved weight gain and feed conversion, enhanced hepatic antioxidant capacity, and optimized intestinal morphology and microbiota by reducing harmful bacteria. GML also increased the total bile acids and conjugated bile acids. In conclusion, dietary 1.0 g/kg GML effectively improves the growth performance and intestinal health in *M. albus*, providing a safe feed additive for healthy aquaculture.

## 1. Introduction

*Monopterus albus* is a commercially important freshwater fish in East Asia, highly valued for its delicate texture and rich nutritional profile, which includes high-quality protein, n-3 polyunsaturated fatty acids, and trace elements such as zinc and selenium [1]. With the rising market demand, the aquaculture output in China exceeded 348,000 tons in 2024 [2]. However, high-lipid diets often induce liver lipid deposition and intestinal dysfunction, which seriously affect the healthy culture of *M. albus*. A typical representative of such metabolic abnormalities is white liver syndrome, which is fundamentally a lipid metabolism disorder triggered by excessive accumulation of triglycerides in hepatocytes [3]. This condition not only compromises liver function but also disrupts intestinal structural integrity, leading to intestinal dysfunction and establishing a pathological interplay along the liver–gut axis [4]. The connection between hepatic lipid metabolic dysfunction and intestinal damage is mechanistically bridged by two key factors: bile acids and gut-derived endotoxins. On one hand, nutrient overload and environmental stressors activate key genes involved in hepatic triglyceride synthesis, leading to fat deposition in hepatocytes and elevated serum triglycerides [5]. This process is mediated by peroxisome proliferator-activated receptor alpha (PPARα) regulating sterol regulatory element-binding protein 1c (SREBP1c) [6], which subsequently influences intestinal lipogenic enzyme activity and exacerbates systemic lipid accumulation [7]. Hepatic lipid overload reduces bile acid synthesis and secretion, impairing intestinal lipid absorption, disrupting mucosal structure, increasing permeability, and allowing for endotoxin translocation [8,9]. On the other hand, these translocated endotoxins, such as lipopolysaccharide (LPS), activate the hepatic TLR4/NF-κB pathway, promoting pro-inflammatory cytokines including interleukin-6 (IL-6) and tumor necrosis factor-alpha (TNF-α) [10]. This liver inflammation further suppresses bile acid synthesis, creating a self-reinforcing vicious cycle along the gut–liver axis. Concurrently, decreased expression of intestinal tight junction proteins (e.g., zonula occludens-1 (ZO-1) and occludin) increases intestinal permeability, exacerbating endotoxin translocation [11]. Thus, bile acids and endotoxins serve as the core mechanistic links that transform parallel liver and intestinal pathologies into an interconnected gut–liver axis. Such a vicious cycle intensifies liver–gut disorders and constrains industry development. Therefore, developing novel feed additives that are capable of modulating both liver and intestinal functions, potentially by restoring bile acid homeostasis or reducing endotoxin translocation, represents a critical direction for the healthy farming of *M. albus*.

Glycerol monolaurate (GML) is a medium-chain fatty acid triglyceride (MCTs) that is naturally present in breast milk, coconut oil, etc., which possesses multiple biological activities, including antibacterial, antiviral, metabolic regulatory, and anti-inflammatory properties, with a low tendency to induce bacterial resistance [12,13]. Recognized as a “Generally Recognized as Safe” (GRAS) substance by the United States Food and Drug Administration (U.S. FDA), GML demonstrates considerable potential as a feed additive, and the antibacterial mechanism involves the insertion of lauric acid chains into bacterial cell membranes, leading to membrane potential collapse, content leakage, and eventual bacterial death [14]. Metabolically, GML upregulates the expression of hepatic carnitine palmitoyltransferase 1 (CPT1), promotes β-oxidation of long-chain fatty acids, inhibits lipogenesis, and reduces triglyceride deposition [15]. In terms of inflammation regulation, GML suppresses LPS-induced reactive oxygen species (ROS) production and NF-κB pathway activation, thereby decreasing the transcription and release of inflammatory cytokines [16]. As a novel feed additive, GML demonstrated clear efficacy in livestock and poultry production, which increased the average daily feed intake and body weight in broilers, improved jejunal villus height, regulated the expression of tight junction protein-related genes, and enhanced antioxidant capacity [17,18], while it also improved the feed conversion rate and growth performance in weaned lambs while modulating rumen microbial community structure [19]. Furthermore, GML enhanced the intestinal structure in piglets, inhibited the proliferation of harmful bacteria, and helped to prevent diseases such as necrotic enteritis [20]. Dietary supplementation with GML modified the milk fatty acid profile in mid-lactation Holstein cows [21]. In recent years, the application of GML in aquatic animals has gained increasing attention. Studies have shown that GML improved the growth performance and modulated gut microbiota in Chinese mitten crabs [22], enhanced the flesh quality of large yellow croakers (*Larimichthys crocea*) [23], increased the survival rate and enhanced the viral resistance of Pacific white shrimp (*Litopenaeus vannamei*) [24], and upregulated the expression of growth- and immune-related genes while improving the intestinal mucosal structure and microbiota composition in juvenile blackhead seabream (*Acanthopagrus schlegelii*) [25].

Although GML has shown beneficial potential in various aquatic animals, systematic research is still lacking for *M. albus*, a species with unique physiological characteristics. In particular, the appropriate dietary supplementation level of GML, its specific regulatory mechanism underlying the liver–gut axis, and its preventive and control effects against common diseases such as enteritis and white liver syndrome remain unclear. Therefore, the present study was conducted to systematically evaluate the effects of dietary GML on growth performance, bile acid metabolism, and intestinal health in *M. albus*. The results will provide a theoretical basis and practical guidance for the scientific application of GML in the green and healthy aquaculture of this species.

## 2. Materials and Methods

### 2.1. Experimental Materials and Diet Formulation

Uniform-sized *M. albus* (initial body weight: 25.0 ± 2.0 g) were obtained from the Zhuanghang Comprehensive Experimental Station of Shanghai Academy of Agricultural Sciences. A total of 225 eels were randomly divided into three groups, with three replicates per group. The experiment was conducted in nine plastic tanks (0.5 m × 0.5 m × 0.5 m), with 25 eels per tank. Prior to the formal experiment, the eels were acclimated in dedicated plastic tanks for one week to adapt to the rearing environment, with stable water quality and quiet conditions maintained during this period. During the 60-day feeding trial, water temperature was controlled at 26 ± 2 °C, dissolved oxygen > 4 mg/L, pH 6.5–7.4, ammonia nitrogen ≤ 0.02 mg/L, and nitrite ≤ 0.1 mg/L. Approximately one-third of the total water volume was replaced every two days. Residual feed and feces were removed daily, feeding behavior was observed and recorded, and water quality parameters were monitored regularly to ensure stable and suitable rearing conditions. All experimental procedures were approved by the Animal Experimental Ethics Committee of Shanghai Academy of Agricultural Sciences (Approval No. SAASXM0625010).

The GML (purity ≥ 95%) used in this study was purchased from Hangzhou Kangyuan Food Technology Co., Ltd. (Hangzhou, China). Three experimental diets were formulated: a basal diet without GML (control group, CON), a basal diet supplemented with 0.5 g/kg GML (GML0.5 group), and a basal diet supplemented with 1.0 g/kg GML (GML1 group). The formulation and proximate nutritional composition of the basal diet are presented in Table 1. All feed ingredients were ground through a 60-mesh sieve, thoroughly mixed, packed in sealed bags, and stored at 4 °C until use.

### 2.2. Sample Collection

At the conclusion of the feeding trial, *M. albus* were fasted for 24 h prior to sampling. Fish from each tank were counted and group-weighed. Six fish were randomly selected from each replicate tank and anesthetized with MS-222 (50 mg/L) (Aldrich Chemical Company, Milwaukee, WI, USA). The standard length, body weight, visceral weight, and liver weight were measured individually for each sampled fish to calculate the viscerosomatic index (VSI), hepatosomatic index (HSI), and condition factor (CF). Blood was collected via the caudal vein method and transferred into 1.5 mL sterile centrifuge tubes. After standing at 4 °C for 24 h, the blood samples were centrifuged at 4000 rpm for 10 min in a refrigerated high-speed centrifuge (Thermo Fisher Scientific (China) Co., Ltd., Shanghai, China). The resulting serum supernatant was collected and stored at −80 °C. Intestinal tissue samples were collected from six randomly selected fish per replicate. A segment of the intestine was fixed in paraformaldehyde for subsequent histological sectioning and analysis. From another six randomly selected fish per replicate, the intestinal contents were aseptically collected into 2 mL centrifuge tubes and immediately frozen at −80 °C for intestinal microbiota analysis. The remaining fish from each tank were dissected to collect intestines and liver, which were stored at −80 °C for the determination of enzymatic activities. All tissue collection and handling procedures were performed on a sterile ice tray to maintain sample integrity.

Samples in the CON group were labeled as CON_1, CON_2, CON_3, CON_4, CON_5, and CON_6, respectively. Samples in the GML0.5 group and GML1 group were labeled as GML0.5_1 to GML0.5_6 and GML1_1 to GML1_6, respectively.

### 2.3. Indicator Measurement

#### 2.3.1. Growth Performance and Organ Indices

The weight gain rate (WGR), feed conversion ratio (FCR), VSI, HSI, and CF are calculated according to the following formulas.

WGR (%) = ((Wm − Wc))/Wc × 100%

FCR (g/g) = Total feed intake (g)/(Wm − Wc)

VSI (%) = Wv/Wm × 100%

HSI (%) = Wh/Wm × 100%

CF (%) = Wm/L^3^ × 100%

In the formula, Wm represents the final total weight (g), Wc represents the initial total weight (g), Wv represents the total visceral weight (g), Wh represents the liver weight of the fish (g), and L represents the body length of the fish (cm).

#### 2.3.2. Determination of Serum Biochemical Indicators

The serum levels of total cholesterol (TC), triglycerides (TG), total protein (TP), albumin (ALB), high-density lipoprotein cholesterol (HDL-C), and low-density lipoprotein cholesterol (LDL-C) were determined. The assays were performed following the instructions of commercial kits (Jiangsu Aidisheng Biotechnology Co., Ltd., Yancheng, China; Catalog Nos.: TC: ADS-W-ZF014, TG: ADS-F-ZF024-96, TP: ADS-W-D008, ALB: ADS-W-DB006, HDL-C: ADS-W-D011, LDL-C: ADS-W-D012) (Jiangsu Aidisheng Biotechnology Co., Ltd., Yancheng, China), with absorbance readings being measured using a microplate reader.

#### 2.3.3. Determination of Immune and Antioxidant Indicators

The activities of acid phosphatase (ACP), alkaline phosphatase (AKP), superoxide dismutase (SOD), and the level of malondialdehyde (MDA) in liver tissue were analyzed. For each assay, approximately 0.1 g of liver tissue was homogenized in 1 mL of physiological saline using a mechanical homogenizer. The homogenization was performed in a cryogenic tissue grinder at 60 Hz for 120 s. Following homogenization, the grinding beads were removed, and the homogenate was centrifuged at 12,000 rpm for 15 min in a refrigerated high-speed centrifuge to obtain the supernatant. The resulting supernatant was then assayed according to the protocols provided with the respective commercial kits (Jiangsu Aidisheng Biotechnology Co., Ltd., China; Catalog Nos.: ACP: ADS-F-ZM002-48; AKP: ADS-W-ZM003; SOD: ADS-W-KY011; MDA: ADS-W-YH002). Absorbance readings were obtained using a microplate reader to determine the final enzymatic activities and MDA concentration (Beijing Zhongliwan Biotechnology Co., Ltd., Beijing, China).

#### 2.3.4. Determination of Bile Acid Content

The analysis of bile acid levels was conducted by Bio-technique (Shanghai Bio-technique Biomedical Technology Co., Ltd., Shanghai, China). The serum bile acid profile of *M. albus* was analyzed using targeted metabolomics based on liquid chromatography–mass spectrometry (LC-MS). For metabolite extraction, a precise volume of 100 μL of serum sample was transferred into an EP tube, to which 400 μL of extraction solvent was added. Corresponding amounts of standard substances were accurately weighed and dissolved to prepare individual stock solutions at a concentration of 1 mg/mL. Appropriate aliquots of these stock solutions were then combined to prepare a mixed standard working solution. Qualitative and quantitative detection were performed using an LC-MS system (model 7890-5975C, Agilent Technologies, Santa Clara, CA, USA). A standard calibration curve was constructed by plotting the peak area ratio of each analyte to its corresponding internal standard against the known concentration of the analyte. The concentration of bile acids in the serum samples was subsequently calculated by substituting the measured peak area ratio (analyte to internal standard) from each sample into the linear regression equation derived from the standard curve.

#### 2.3.5. Preparation and Observation of Intestinal Tissue Sections

Intestinal tissue samples were dehydrated and embedded in paraffin. Sections were stained using the hematoxylin and eosin (HE) staining method for histological examination. The stained sections were observed and photographed under a research-grade upright microscope (Nikon, Tokyo, Japan). Using ImageJ software (Version 1.54r; National Institutes of Health, Bethesda, MD, USA), key morphometric parameters of the intestinal cross-sections were measured, including villus height (VH), villus width (VW), and muscular thickness (MT). The measurement criteria were as follows: VH was defined as the distance from the villus tip to the villus base (villus–crypt junction); VW was measured as the width at the midpoint of the villus; and MT was defined as the thickness of the longitudinal muscle layer from the inner to the outer boundary. Six random fields per section were analyzed, and the mean values were calculated.

#### 2.3.6. Intestinal Microbiota

Sequencing of the intestinal microbiota was conducted by Bio-technique (Shanghai Bio-technique Biomedical Technology Co., Ltd., Shanghai, China). Total DNA was extracted from the collected intestinal content samples according to the instructions of the DNA extraction kit. The composition of the intestinal microbial community was determined by analysis on the Illumina MiSeq PE300 platform (Illumina, San Diego, CA, USA). Using UPARSE software (version 11), sequences were clustered into Amplicon Sequence Variants (ASVs) based on a 97% similarity threshold. Principal Coordinate Analysis (PCoA) based on ASV composition was performed to differentiate the microbial profiles among different experimental groups. A total of six samples per group were sequenced and all were included in the final analysis. All analyses of the microbiota data were carried out on the free online platform of the Biotree Lims2 cloud system (https://biotree.lims2.com/home, accessed on 13 May 2026).

### 2.4. Data Processing and Analysis

The experimental data were initially collated using Excel 2019 software. One-way analysis of variance (one-way ANOVA) was performed using Prism 9.5.1, followed by Tukey’s multiple comparison test when significant differences were detected. Homogeneity of variance was tested using SPSS 26.0 (IBM, Armonk, NY, USA). One-way ANOVA followed by Tukey’s test was conducted for further group comparisons. Statistical significance was indicated as follows: *p* < 0.05 (*), *p* < 0.01 (**), and *p* < 0.001 (***).

## 3. Results

### 3.1. Growth Performance

As shown in Table 2, compared with the CON group, the WGR of the GML1 group significantly increased by 8.75% (*p* < 0.05), whereas the GML0.5 group showed a 2.85% increase that was not statistically significant (*p* > 0.05). Similarly, the FCR of the GML1 group significantly decreased by 8.16% (*p* < 0.05), while the GML0.5 group exhibited a 3.06% decrease without a significant difference (*p* > 0.05). With the increase in GML concentration, the VSI, HSI and CF in the GML1 group were significantly higher than those of the CON group (*p* < 0.05).

### 3.2. Serum Biochemical Parameters

As presented in Table 3, compared with the CON group, dietary GML supplementation had no significant effects on the contents of TC and TG (*p* > 0.05). The levels of TP and ALB were significantly decreased in the GML-treated groups (*p* < 0.05). The HDL-C content showed an upward trend in the GML0.5 group, while LDL-C content tended to decrease in both the GML0.5 and GML1 groups, but none of these changes were statistically significant (*p* > 0.05).

### 3.3. Immune and Antioxidant Parameters

The immune and antioxidant parameters measured in liver tissue are presented in Table 4. Compared with the CON group, the ACP content in the GML0.5 group was significantly decreased (*p* < 0.05), while no significant difference was observed in the GML1 group (*p* > 0.05). The AKP content was significantly reduced in both the GML0.5 and GML1 groups (*p* < 0.05). Regarding antioxidant capacity, after GML supplementation, the MDA content was significantly decreased by 24.80% and 38.15% in the GML0.5 and GML1 groups, respectively (*p* < 0.05).

### 3.4. Bile Acid Contents

**Figure 1:**
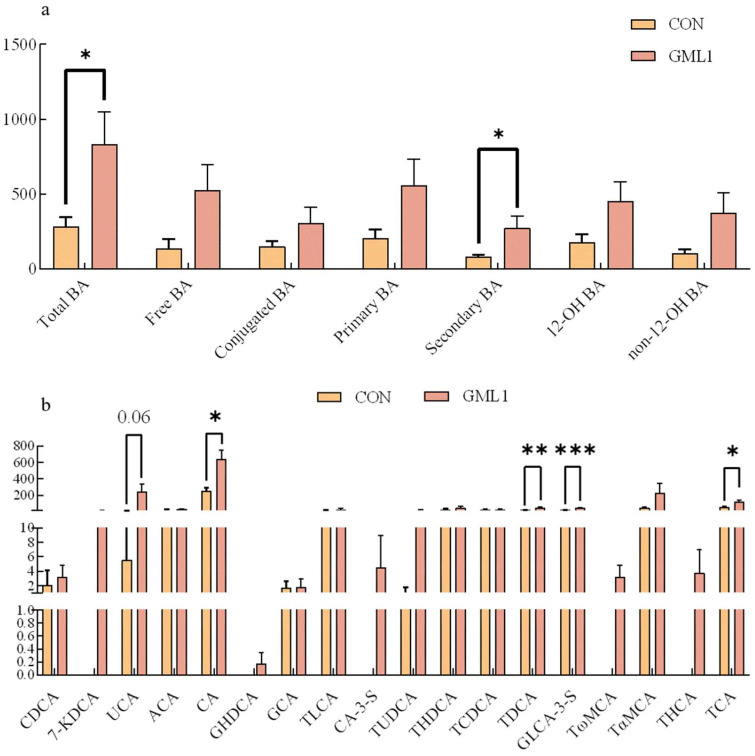
(**a**) The effect of GML on the content of individual bile acids. (**b**) The effect of GML on the total bile acid content. *p* < 0.05 (*), *p* < 0.01 (**), and *p* < 0.001 (***).

Compared with the CON group (Figure 1), the contents of total bile acid (BA) and secondary bile acids in the GML1 group were significantly increased (*p* < 0.05). Further analysis (Figure 1) revealed that the blood concentrations of cholic acid (CA), taurodeoxycholic acid (TDCA), glycochenodeoxycholic acid 3-sulfate (3S-GLCA) and taurocholic acid (TCA) in the GML1 group were significantly elevated (*p* < 0.05), while the ursodeoxycholic acid (UCA) content exhibited an upward trend (0.05 < *p* < 0.1).

### 3.5. Intestinal Histological Morphology

**Figure 2:**
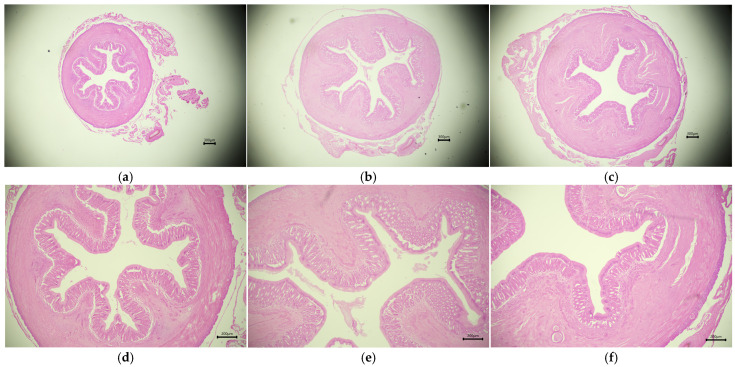
Effects of GML on intestinal histomorphology of *M. albus*. (**a**–**c**) The intestinal histological structures of the CON, GML0.5, and GML1 groups under 40× magnification, respectively. (**d**–**f**) The intestinal histological structures of the CON, GML0.5, and GML1 groups under 100× magnification, respectively.

The hematoxylin–eosin (H&E)-stained intestinal sections of *M. albus* are presented in Figure 2. Histomorphological observation revealed that the intestinal villi in the CON group were shorter with a thinner muscular thickness, whereas the villus height in the GML0.5 and GML1 groups were longer, more neatly arranged, and accompanied by a thicker muscular thickness. As shown in Table 5, the villus height (VH), villus width (VW), and muscular thickness (MT) in the GML0.5 and GML1 groups were significantly higher than those of the CON group (*p* < 0.05).

### 3.6. Intestinal Microbiota

**Figure 3:**
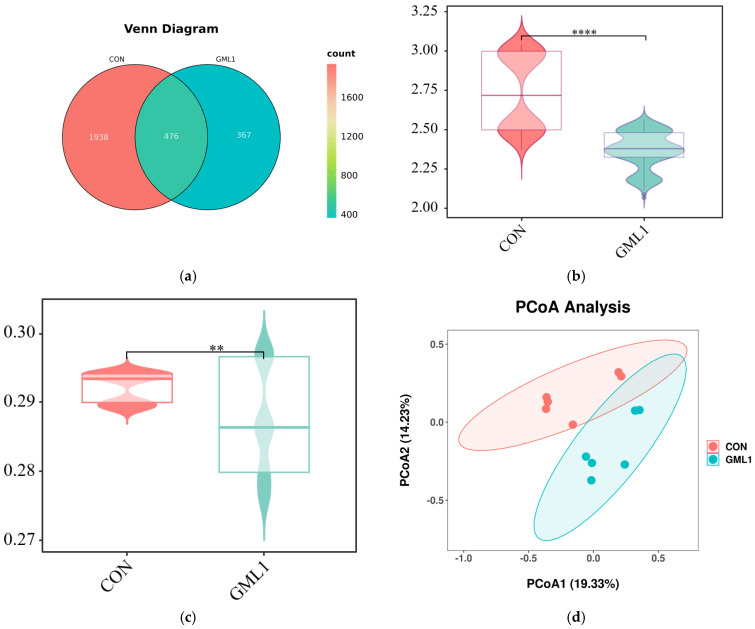
Effects of GML on the α-diversity and β-diversity of intestinal microbiota. (**a**) The Venn diagram of intestinal microbial species in the CON and GML groups. (**b**,**c**) The α-diversity indices (Chao1 and Simpson indices) of intestinal microbiota in the CON and GML groups, respectively. (**d**) The principal coordinate analysis (PCoA) of amplicon sequence variants (ASVs) based on 16S rRNA gene sequencing. *p* < 0.01 (**), and *p* < 0.0001 (****).

**Figure 4:**
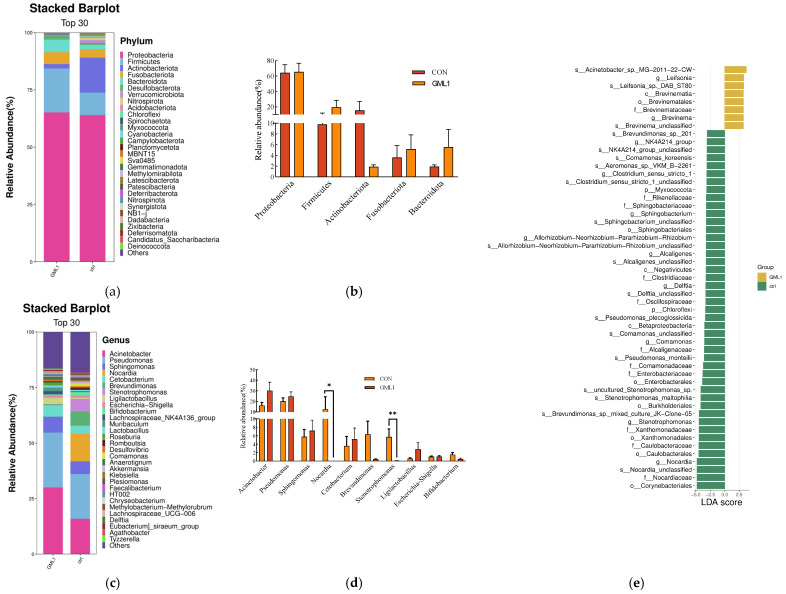
Analysis of the effects of GML on the intestinal microbiota composition of *M. albus.* (**a**) The intestinal microbiota composition at the phylum level in the CON and GML groups. (**b**) The variations in the top 5 most abundant phyla of the intestinal microbiota between the CON and GML groups. (**c**) The intestinal microbiota composition at the genus level in the CON and GML groups. (**d**) The variations in the top 10 most abundant genera of the intestinal microbiota between the CON and GML groups. (**e**) The linear discriminant analysis effect size (LEfSe) from the phylum to the genus level (LDA > 3.0). *p* < 0.05 (*), *p* < 0.01 (**).

As shown in Figure 3a, a total of 2781 amplicon sequence variants (ASVs) were detected. Among them, 476 ASVs were shared between the CON and GML1 groups, while the numbers of unique ASVs in the CON and GML1 groups were 1938 and 367, respectively. As presented in Figure 3b,c, compared with the CON group, the Chao1 index of the GML1 group was significantly decreased, whereas the Simpson index was significantly increased (*p* < 0.05). As indicated in Figure 4d, principal coordinate analysis (PCoA) showed a moderate but significant separation of microbial community composition between the GML1 group and the CON group. As shown in Table 6, non-parametric multivariate analysis of variance (Adonis) was used to evaluate the explanatory power of different grouping factors on sample differences. An intergroup *R*^2^ value of 0.2 indicated that the grouping accounted for 20% of the total variation; an *F* value of 2.49 suggested that the intergroup variation was 2.49 times the intragroup variation, demonstrating a significant difference between groups (*p* < 0.05).

At the phylum level, the intestinal microbiota of *M. albus* was primarily composed of *Proteobacteria*, *Firmicutes*, *Actinobacteriota*, *Fusobacteriota*, and *Bacteroidota* (Figure 4a). Compared to the CON group, the GML1 group showed trends of increased relative abundance of *Firmicutes*, *Fusobacteriota*, and *Bacteroidota*, and a decreased trend in *Actinobacteriota*, but none of these changes reached statistical significance (*p* > 0.05, Figure 4b). At the genus level, the dominant taxa included *Acinetobacter*, *Pseudomonas*, *Sphingomonas*, *Nocardia*, and *Cetobacterium* (Figure 4b,c). Notably, the abundances of *Nocardia* and *Stenotrophomonas* were significantly reduced in the GML1 group compared to the CON group (*p* < 0.05).

As illustrated in Figure 4e, the linear discriminant analysis effect size (LEfSe) revealed that the dominant microbial taxa in the CON group were *Corynebacteriales*, *Nocardiaceae*, *Nocardia*_unclassified, and *Nocardia*, whereas in the GML1 group, the predominant taxa were identified as *Acinetobacter*_sp._MG-2011-22-CW, *Leifsonia*, *Leifsonia*_sp._DAB ST80, *Brevinematia*, *Brevinematales*, *Brevinemataceae*, and *Brevinema*.

**Figure 5:**
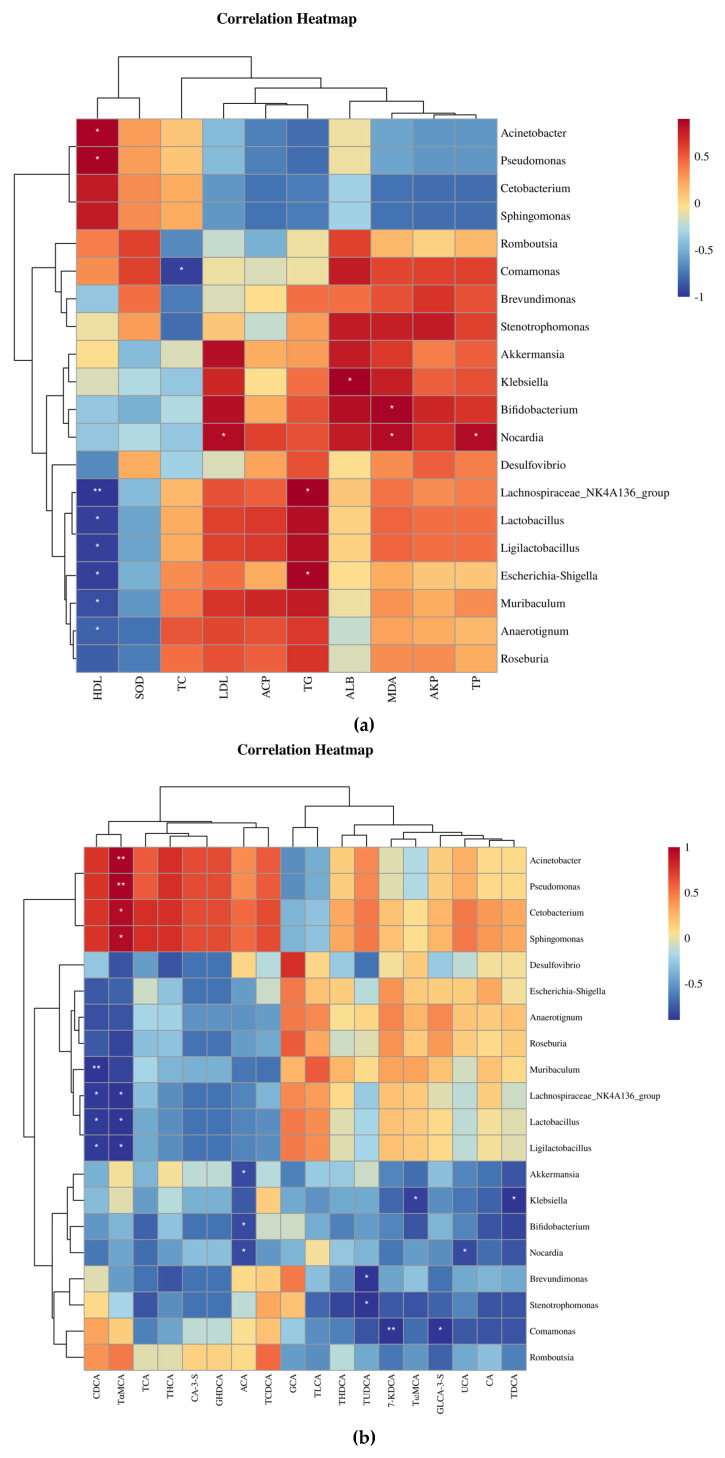
Correlation analysis between gut microbial abundance, biochemical indicators and bile acids. Note: (**a**) shows the correlation analysis between intestinal microbial abundance and biochemical indices, and (**b**) shows the correlation analysis between intestinal microbial abundance and bile acids. Red and blue represent extremely significant positive and negative correlations, respectively. * indicates that *p* < 0.05, representing a significant difference and ** indicates that *p* < 0.01, representing an extremely significant difference. The statistical method used in this study was Spearman’s correlation analysis.

Spearman correlation analysis between the top 20 abundant genera and serum biochemical and hepatic immune markers (Figure 5a) showed that genera such as *Acinetobacter*, *Pseudomonas*, and *Cetobacterium* were positively correlated with HDL-C, SOD, and TC, and negatively correlated with LDL-C, TG, MDA, AKP, and ACP, suggesting potential roles in modulating lipid metabolism and inflammatory responses. Conversely, *Akkermansia* and *Roseburia* were positively correlated with AKP, ACP, TP, and TG, indicating their potential association with enhanced intestinal barrier function and nutrient absorption. *Nocardia* exhibited positive correlations with LDL-C and MDA, implying its potential involvement in promoting oxidative stress and inflammation. Spearman correlation analysis was performed between the top 20 genera in relative abundance and bile acids (Figure 5b). Chenodeoxycholic acid was negatively correlated with *Muribaculum*, *Lactobacillus*, and other genera (*p* < 0.05), suggesting that GML may inhibit primary bile acid synthesis or the enterohepatic circulation. Tauro-α-muricholic acid was negatively correlated with *Lactobacillus*, *Ligilactobacillus*, and other genera (*p* < 0.05), and positively correlated with *Acinetobacter*, *Pseudomonas*, *Cetobacterium*, and *Sphingomonas* (*p* < 0.05), indicating that beneficial bacteria such as *Cetobacterium* enriched by GML can promote the synthesis of conjugated bile acids and improve the intestinal lipid absorption efficiency. Allocholic acid was negatively correlated with *Nocardia*, *Bifidobacterium*, and *Akkermansia* (*p* < 0.05). Several bile acids, including tauroursodeoxycholic acid, were significantly negatively correlated with opportunistic pathogens such as *Nocardia* (*p* < 0.05), indicating that GML can inhibit the proliferation of harmful bacteria and thereby alleviate their inhibitory effect on bile acid metabolism.

## 4. Discussion

### 4.1. Effects of GML on the Growth Performance of M. albus

The results of this study demonstrated that dietary supplementation with GML exerted a significant regulatory effect on the growth performance of *M. albus*. We found that increasing dietary GML supplementation led to a significant increase in WGR and a significant decrease in FCR, with the most prominent effects observed in the GML1 group, indicating that an appropriate dose of GML could effectively enhance the growth rate and feed utilization efficiency of *M. albus*. Organ indices are important indicators reflecting the growth and development status and functional balance of visceral organs in organisms. The significantly higher VSI, HSI, and CF in the GML1 group suggested that GML might affect the growth and metabolic processes of *M. albus* by regulating the pattern of fat accumulation and distribution in the body and optimizing the flow direction of nutrient metabolism, thus providing phenotypic evidence for further exploration of its physiological regulatory mechanism.

The existing studies have confirmed that GML exhibits potential in improving the performance of both livestock, poultry, and aquatic animals. In the field of livestock and poultry production, Liu et al. [17] found that GML supplementation significantly increased the average daily feed intake (ADFI) and final body weight (BW) of broilers aged 28–56 days, thereby improving the production performance of broilers. The underlying mechanism might be related to the ability of dietary GML to enrich acid-producing bacteria such as *Lachnospiraceae* and *Bifidobacteriaceae*, inhibit harmful bacteria, and stimulate the production of short-chain fatty acids (SCFAs), thus improving intestinal morphology and health. However, other studies pointed out that GML supplementation at doses of 0.9 g/kg and 1.2 g/kg increased the feed intake of broiler chicks, but it had no significant effect on their BW, BWG, and FCR [26]. This indicated that the promoting effect of GML on the digestibility and growth performance of broiler chicks was mainly manifested by inhibiting the proliferation of pathogenic bacteria.

It is important to note that physiological characteristics and living environments differ between aquatic animals and livestock. Aquatic animals are mostly poikilothermic and inhabit aquatic environments, and their intestinal structures also differ from those of terrestrial livestock. These factors may influence the absorption and efficacy of GML. Nevertheless, the growth-promoting effects of GML in aquatic animals have been partially validated. Li et al. [27] reported that dietary supplementation with GML of 1.8 g/kg significantly improved the WGR of the hybrid grouper (*Epinephelus fuscoguttatus* ♀ × *Epinephelus lanceolatus* ♂), consistent with the growth response trend observed in *M. albus* in this study. In a 42-day feeding trial with Pacific white shrimp (*Penaeus vannamei*), the GML-supplemented group showed a significantly increased weight gain rate and a significantly reduced feed conversion ratio [28], further supporting the potential of GML to improve growth performance in both crustaceans and fish.

As a typical representative of medium-chain fatty acid monoglycerides, GML possesses anti-inflammatory and antibacterial biological properties [29]. Its molecular structure enables rapid penetration of cell membranes, and it can be directly utilized as an energy source after digestion, providing an energetic foundation for growth and metabolism [30]. This is one of the key mechanisms through which GML exerts its growth-promoting effects. Furthermore, the biological effects of GML exhibit concentration-dependent characteristics. Mo et al. [31] found that GML had a concentration-dependent effect, and high-dose GML supplementation at 1.6 g/kg could promote the growth and increase the weight gain rate of mice (*Mus musculus*), which was consistent with the dose-effect trend of GML observed in this study. Rimoldi et al. [32] reported that dietary supplementation with medium- and short-chain fatty acid monoglycerides could increase the abundance of beneficial bacteria such as *Lactobacillus* in the intestine of gilthead seabream (*Sparus aurata*), thereby significantly improving feed efficiency. Hanczakowska et al. [33] also noted that medium-chain fatty acids reduced the abundance of intestinal pathogens and significantly increased the weight gain rate in piglets. These studies collectively demonstrate that GML exerts dual effects on most animals by promoting growth and optimizing intestinal microbiota, and the regulatory effects are likely achieved through the synergistic action of multiple physiological pathways.

### 4.2. Effects of GML on Serum Biochemical Parameters of M. albus

Blood serves as a central carrier for substance transport and the regulation of internal homeostasis, playing a crucial role in maintaining the physiological functional balance of fish. Serum biochemical parameters in fish directly reflect the nutrient absorption efficiency, metabolic homeostasis, and pathophysiological status [34], and are important biological markers for assessing immune function and overall health. Therefore, analyzing changes in these indicators helps elucidate the physiological regulatory mechanisms of GML in *M. albus*.

Lipid metabolism represents a central process in the organism’s energy metabolism, and blood serves as the primary medium for lipid transport. The serum concentrations of TC and TG directly reflect the overall intensity of lipid synthesis, degradation, and transport within the body [35]. In cholesterol transport, LDL-C and HDL-C function as key carriers for transporting liver-synthesized cholesterol to peripheral tissues. Variations in their levels accurately indicate the dynamic balance of cholesterol metabolism in the organism [36]. We found that dietary GML supplementation led to non-significant trends of increased TC and HDL-C and decreased TG and LDL-C in the GML0.5 group. These trends suggest that GML may regulate cholesterol metabolism by affecting VLDL-C-mediated transport or shifting the balance between cholesterol synthesis and excretion. Given that bile acids are the primary end products of cholesterol catabolism, this regulatory effect is likely mediated through bile acid metabolism. Specifically, enhanced bile acid synthesis would promote cholesterol clearance and lower LDL-C, whereas impaired bile acid metabolism would lead to cholesterol accumulation [37]. Thus, the observed lipid profile trends, although not statistically significant, provide preliminary evidence for a GML, bile acid and cholesterol regulatory axis in *M. albus*.

The regulatory effect of GML on serum lipid parameters exhibits significant species specificity and dose dependence. Liu et al. [17] reported that supplementing 0.45 g/kg and 0.6 g/kg GML to the diet of yellow-feathered broilers exerted no significant effects on the serum contents of TG, LDL-C, and HDL-C, with only an increasing trend in TG content observed. These findings are consistent with the serum changes observed in *M. albus* in the present study. In contrast, research on Pacific white shrimp showed that GML significantly increased the serum’s TC, TG, and LDL-C levels while markedly reducing the HDL-C concentration [28]. These discrepancies may arise from differences in lipid metabolic pathways and the efficiency of GML absorption and utilization between avian and aquatic species, and the underlying mechanisms warrant further elucidation.

Serum TP serves as a core indicator of the organism’s protein metabolic capacity, with its concentration directly linked to amino acid absorption, utilization, and protein synthesis efficiency. We found that GML supplementation significantly reduced serum TP and ALB levels in *M. albus* compared to the control group, which suggests that GML may influence amino acid transport and hepatocyte protein synthesis function, thereby altering the protein metabolic balance in *M. albus*. However, Lin et al. [15] reached an opposite conclusion in juvenile golden pompano (*Trachinotus ovatus*), where dietary supplementation with 0.05%, 0.10%, and 0.15% GML significantly increased serum TP levels. The authors proposed that GML enhances metabolic activity, thereby promoting protein synthesis and nitrogen retention. These contradictory findings may be attributed to the differential effects of GML on intestinal health and hepatocyte function across fish species. The intestine, as the primary site for amino acid absorption, determines absorption efficiency through mucosal integrity and digestive enzyme activity [38]. Meanwhile, the liver, as the central organ for protein synthesis, influences the production of secretory proteins such as ALB [39]. Therefore, the downregulatory effects of GML on serum TP and ALB in *M. albus* may indirectly reflect its impact on the intestinal morphological structure, intestinal absorption function, or hepatocyte metabolic activity. Further investigation is required to elucidate these mechanisms through the lens of intestinal health and liver function.

### 4.3. Effects of GML on the Immune and Antioxidant Capacity of M. albus

The liver of *M. albus* plays an indispensable role in various physiological processes, including substance metabolism [40], detoxification [41], immune defense [42], and nutrient storage, all of which are crucial for maintaining normal growth, development, and overall health. The differential changes in ACP and AKP activities suggest targeted regulation of liver function by GML. ACP is mainly located in hepatic lysosomes and enhances the efficiency of pathogen clearance by modifying the surface recognition system of foreign substances [43]. These two enzymes primarily reflect the immune status, rather than direct antioxidant capacity. AKP is a key immune factor involved in lipid metabolism and energy balance [44]. We found that the AKP content was significantly decreased in both GML-supplemented groups, which suggests that GML may reduce excessive immune consumption mediated by ACP via inhibiting ACP activity, and moderately downregulated AKP activity to alleviate metabolic load, thereby maintaining hepatic function stability. Zhang et al. [45] found that 0.50–0.75 g/kg GML significantly increased the AKP activity in largemouth bass (*Micropterus salmoides*), confirming that GML exerts a universal regulatory effect on AKP in fish. In contrast, Li et al. [27] reported that in the hybrid grouper, ACP and AKP activities initially increased and then decreased with increasing dietary GML levels, peaking at 1.8 g/kg. The enzyme activities declined at high doses due to excessive metabolic load, which is logically consistent with the non-significant change in ACP observed in the GML1 group of the present study.

Regarding antioxidant capacity, SOD is a critical enzyme that is responsible for scavenging superoxide anion radicals [46], and the activity is directly linked to the extent of cellular oxidative damage. MDA, as a terminal product of lipid peroxidation [47], serves as a core marker for assessing oxidative stress. We observed that MDA levels were significantly reduced in the GML-supplemented groups, whereas SOD activity showed no significant change. This pattern suggests that GML may mitigate liver inflammation and oxidative stress by optimizing the intestinal microbiota structure and reducing endotoxin translocation, rather than by directly enhancing SOD activity [26]. Additionally, the decline in MDA indicates reduced lipid peroxidation, thereby protecting the integrity of hepatocyte membranes. These findings are consistent with the conclusions of Lin et al. [15] in juvenile golden pompano. Together, the reduced MDA levels and modulated immune parameters in this study further verify the protective effect of GML on liver function in fish.

### 4.4. Effects of GML on Bile Acid Synthesis and Metabolism in M. albus

Bile acids, the core lipid components of bile, are synthesized exclusively from cholesterol in the liver. Their composition and concentration are directly regulated by synthesis, secretion, and enterohepatic circulation [48]. As a carnivorous-leaning freshwater fish, *M. albus* typically consumes diets high in crude lipid [5]. Efficient digestion and absorption of dietary lipids depend heavily on the emulsifying function of bile acids, placing higher demands on the rate of bile acid synthesis and the efficiency of enterohepatic circulation. Consequently, the total amount and composition of the bile acid pool directly determine lipid metabolic homeostasis [49]. Moreover, *M. albus* possesses a unique gastrointestinal structure characterized by a short intestine, absence of a stomach, and monogastric digestion [50]. This physiological adaptation not only requires more efficient secretion and reabsorption of bile acids but also makes the bile acid metabolism process more susceptible to modulation by exogenous substances via alterations in the intestinal environment. Consistent with this susceptibility, our results showed that GML supplementation significantly modulated bile acid profiles.

In this study, dietary GML supplementation significantly increased the total serum bile acid levels, with ursodeoxycholic acid, cholic acid, tauro-α-muricholic acid, and taurocholic acid being the predominant species. The GML1 group also exhibited elevated serum cholesterol levels. GML can be hydrolyzed to free lauric acid for rapid absorption via the portal vein [51] or emulsified by bile acids and absorbed via chylomicrons into the lymphatic system [52]. The observed bile acid and cholesterol profiles suggest that GML accumulation on the intestinal mucosal surface may enhance the emulsifying efficiency of bile acids for high-fat diets, accelerate lipid hydrolysis, and activate negative feedback regulation via the gut–liver axis, thereby promoting hepatic cholesterol conversion to primary bile acids [13,53].

Considering the neutral intestinal pH environment of *M. albus* [54], conjugated bile acids exhibit significantly higher emulsifying activity than their free counterparts under such conditions [55]. The significant increase in tauro-α-muricholic acid in the GML1 group suggests that this conjugated bile acid enhances the overall emulsifying capacity, improving lipid-derived energy absorption efficiency and contributing to the observed decrease in FCR and increase in WGR. Additionally, tauro-α-muricholic acid may act as a farnesoid X receptor (FXR) antagonist [56], potentially regulating intestinal glucagon-like peptide-1 (GLP-1) secretion, which has been shown to reduce visceral fat deposition and improve lipid metabolism [57].

Notably, the intestinal microbiota plays a crucial supporting role in GML-mediated regulation of bile acid metabolism in *M. albus*. Gut microorganisms can convert primary bile acids into free bile acids via bile salt hydrolase (BSH), which can then be transformed into secondary bile acids through 7α-dehydroxylation [58]. GML may optimize the intestinal microbial structure, reduce BSH activity, decrease the hydrolysis of conjugated bile acids, and help maintain the dynamic balance of the bile acid pool [59]. Conversely, bile acids regulate gut microbiota composition through receptors such as FXR and TGR5, alleviating inhibition of hepatic cholesterol 7α-hydroxylase (CYP7A1), promoting primary bile acid synthesis, increasing the proportion of conjugated bile acids [60], and protecting the liver and intestine from inflammatory damage [61]. Thus, the interaction between gut microbiota and bile acids plays a key role in mitigating metabolic disturbances.

### 4.5. Effects of GML on Intestinal Health of M. albus

The intestine serves as the primary site for nutrient digestion and absorption in *M. albus* and functions as a crucial barrier against pathogen invasion, and the health status directly determines the growth performance and metabolic homeostasis of *M. albus* [62], while the structural integrity of the intestinal microbiota governs host nutrient absorption efficiency and immune defense capabilities [63].

Intestinal structural integrity is a key indicator for evaluating gut health [64]. VH and MT are core parameters reflecting the intestinal physical barrier and nutrient absorption capacity [65]. In this study, the GML-supplemented groups exhibited significantly increased villus height, villus width, and muscular thickness, consistent with the findings of Lin [66] and Li [20]. Histological examination further revealed well-aligned, intact villi with dense muscular layers. These results suggest that GML promotes proliferation and differentiation of intestinal mucosal epithelial cells, increasing villus absorption area and enhancing mechanical support of the muscular layer [67]. The expanded villus surface area improves the digestion and absorption efficiency of dietary proteins and lipids, explaining the significant increase in WGR and decrease in FCR. Moreover, an intact villus structure and dense muscular layer reduce intestinal epithelial leakage, lowering endotoxin translocation into the bloodstream and indirectly alleviating hepatic oxidative stress [68], as evidenced by decreased MDA levels. LEfSe analysis further revealed that the GML1 group was enriched in functional bacteria such as *Leifsonia* and *Brevinema*, which are associated with lipid metabolism and anti-inflammatory responses. In contrast, the CON group was characterized by inflammation-associated taxa, including *Nocardiaceae* and *Corynebacteriales.* These compositional shifts are physiologically relevant, as previous studies have shown that GML can upregulate beneficial bacteria like *Cetobacterium* in zebrafish [69]. *Cetobacterium* produces vitamin B12, which acts as a gut microbial modulator, enhancing inter-microbial interactions and tight junction barrier function, thereby improving host resistance to pathogen infection [70]. As a dominant intestinal bacterium, *Cetobacterium* contributes to maintaining fish health by regulating immune function, intestinal health, and energy metabolism [71].

Correlation analysis provided further insights into the functional relevance of microbiota changes. The abundance of *Cetobacterium* was higher in GML groups and was positively correlated with HDL-C and SOD but negatively correlated with LDL-C and MDA. This pattern suggests that *Cetobacterium* may support intestinal health by modulating glucose and lipid metabolism and enhancing antioxidant capacity [72]. Conversely, *Nocardia* was positively correlated with LDL-C and MDA (*p* < 0.05), indicating that its reduction following GML supplementation may directly lower intestinal oxidative stress and inflammation risk. These correlational links establish a clear bridge between GML-induced microbiota remodeling and host metabolic/immune parameters.

The intestinal microbiota and bile acids engage in bidirectional interactions that critically influence host metabolism [73]. In this study, the GML1 group exhibited significantly elevated levels of total bile acids and functional bile acids, including tauro-α-muricholic acid and ursodeoxycholic acid, which were closely linked to improved intestinal health. Correlation analysis demonstrated that tauro-α-muricholic acid was negatively correlated with *Lactobacillus* and *Ligilactobacillus* but positively correlated with GML-enriched beneficial bacteria such as *Acinetobacter* and *Pseudomonas*. This correlation pattern suggests that GML-enriched beneficial bacteria may promote conjugated bile acid synthesis and enhance intestinal lipid digestion and absorption, thereby establishing a positive feedback loop between beneficial gut microbiota and bile acid metabolism. Previous studies have demonstrated similar microbiota–bile acid interactions. Chen et al. [74] demonstrated that resveratrol could inhibit the ileal FXR-FGF15 axis, increase the abundance of *Lactobacillus* and *Bifidobacterium*, enhance BSH activity, promote the deconjugation of conjugated bile acids, increase fecal bile acid excretion, and ultimately reduce ileal bile acid levels. Ning et al. [75] found that palmatine treatment could maintain intestinal microbiota balance, regulate bile acid metabolism, and reduce blood lipids in rats by mediating the PPARα-CYP7A1 pathway, thereby protecting against hyperlipidemia. Extending these findings, our results suggest that GML, hydrolyzed to lauric acid for rapid absorption, may activate gut–liver axis feedback regulation, promoting the conversion of hepatic cholesterol to primary bile acids. The increased proportion of tauro-α-muricholic acid, which exhibits higher emulsifying activity than free bile acids under the near-neutral intestinal pH conditions of *M. albus*, may further enhance crude fat absorption efficiency—consistent with the observed decrease in FCR [76]. Furthermore, functional bile acids such as allocholic acid were significantly negatively correlated with opportunistic pathogens including *Nocardia*, suggesting that GML-induced increases in bile acids may suppress harmful bacterial proliferation. Meanwhile, the direct antibacterial effect of GML may also relieve pathogen-mediated negative regulation of bile acid metabolism. The significant increase in ursodeoxycholic acid in the GML1 group is particularly noteworthy, as this bile acid possesses well-documented anti-inflammatory properties and is involved in immune and bile acid regulation [76,77].

Intestinal homeostasis relies on the coordination of digestive–absorptive and barrier functions, with bile acids acting as critical signaling molecules linking these processes [78]. Notably, the beneficial changes in the bile acid profile observed in this study closely correspond to immune regulatory responses at the hepatic transcriptomic level. Previous research on immune pathways in the liver of *M. albus* [79] revealed that GML supplementation significantly downregulated pro-inflammatory genes such as *IL12*, *IL18R1*, and *IL21R*, directly inhibiting Th1 cell differentiation and the secretion of IFN-γ. Simultaneously, significant upregulation of *CD49* may enhance immune cell adhesion functions. This differential regulation of gene expression further supports the notion that GML exerts multi-level synergistic effects through the bile acid, gut and liver axis, ranging from local intestinal protection to hepatic immune modulation, collectively strengthening intestinal health and immune defense in the host.

## 5. Conclusions

The findings of this study demonstrate that dietary supplementation with 1.0 g/kg GML achieves multi-dimensional optimization of growth performance, metabolic regulation, and health maintenance in *M. albus*. This inclusion level significantly enhances the weight gain rate, reduces the feed conversion ratio, and improves the viscerosomatic and hepatosomatic indices, thereby effectively increasing feed economic conversion efficiency. Furthermore, GML alleviates hepatic damage by modulating lipid metabolism and strengthening antioxidant capacity while promoting the synthesis of functional bile acids to optimize the enterohepatic circulation. Regarding intestinal health, GML improves intestinal morphological structure, enhances the physical barrier function, and optimizes microbial composition by reducing the abundance of potential pathogens. Collectively, these results indicate that dietary GML exerts beneficial effects on the growth, metabolism, and health status of *M. albus*, and 1.0 g/kg GML shows more favorable effects than 0.5 g/kg under the conditions of this study.

## Figures and Tables

**Table 1:** Composition of the basal diet (air-dried basis, g/kg).

Ingredients	CON	GML0.5	GML1
Fish meal	450.0	450.0	450.0
Soybean meal	180.0	180.0	180.0
α-starch	140.0	140.0	140.0
Wheat flour	75.0	74.5	74.0
Corn gluten meal	65.0	65.0	65.0
Yeast meal	20.0	20.0	20.0
Fish oil	20.0	20.0	20.0
Soybean oil	10.0	10.0	10.0
Premix ^a^	20.0	20.0	20.0
Ca(H_2_PO_4_)_2_	15.0	15.0	15.0
Choline chloride	5.0	5.0	5.0
GML	0.0	0.5	1.0
Total	1000.0	1000.0	1000.0
Nutrient composition ^b^			
Moisture	103.9	103.8	103.1
Crude protein	430.2	432.1	432.2
Crude lipid	72.3	72.8	73.2

Note: ^a^ premix (mg/kg or IU/kg): VA 6000 IU, VD_3_ 3000 IU, VE 300 mg, VK_3_ 45 mg, VB_1_ 40 mg, VB_2_ 60 mg, VB_3_ 300 mg, VB_6_ 30 mg, VB_12_ 5 mg, VC 2300 mg, biotin 3 mg, folic acid 30 mg, inositol 800 mg, thiamine 50 mg, riboflavin 10 mg, niacin 200 mg, pantothenic acid 60 mg, microcrystalline cellulose 3240.47 mg, iodine 50 mg, cobalt 60 mg, copper 340 mg, iron 1500 mg, zinc 650 mg, manganese 520 mg, selenium 12 mg, magnesium 2000 mg, and zeolite powder 5650.85 mg. ^b^ Nutrient levels were measured values.

**Table 2:** Effects of GML on growth performance of *M. albus*.

Index	CON	GML0.5	GML1
IBW (g)	25.00 ± 1.50	25.00 ± 1.70	25.00 ± 1.80
FBW (g)	56.63 ± 1.12	57.53 ± 1.35	59.4 ± 1.01
WGR (%)	126.53 ± 4.46 ^b^	130.13 ± 5.40 ^ab^	137.60 ± 6.34 ^a^
FCR (g/g)	1.96 ± 0.07 ^a^	1.90 ± 0.08 ^ab^	1.80 ± 0.06 ^b^
VSI (%)	5.74 ± 0.56 ^b^	6.72 ± 0.63 ^ab^	6.86 ± 0.26 ^a^
HSI (%)	2.02 ± 0.18 ^b^	1.71 ± 0.09 ^b^	3.84 ± 0.28 ^a^
CF (%)	0.08 ± 0.00 ^b^	0.08 ± 0.01 ^b^	0.10 ± 0.01 ^a^

Note: Abbreviations are defined as follows: IBW refers to average initial body weight; FBW refers to average final body weight; WGR refers to weight gain rate; FCR refers to feed conversion ratio; VSI refers to viscerosomatic index; HSI refers to hepatosomatic index; and CF refers to condition factor. Values with different lowercase letters in the same row differ significantly (*p* < 0.05).

**Table 3:** Effects of GML on serum biochemical parameters of *M. albus*.

Index	CON	GML0.5	GML1
TC (mmol/L)	4.25 ± 0.30	4.50 ± 0.40	4.05 ± 0.25
TG (mmol/L)	0.13 ± 0.05	0.08 ± 0.00	0.09 ± 0.04
TP (g/L)	42.23 ± 4.52 ^a^	28.68 ± 0.92 ^b^	24.78 ± 0.71 ^b^
ALB (g/L)	14.21 ± 0.53 ^a^	12.35 ± 0.74 ^b^	11.77 ± 0.15 ^b^
HDL-C (mmol/L)	2.36 ± 0.13	2.66 ± 0.35	2.24 ± 0.54
LDL-C (mmol/L)	1.24 ± 0.31	1.01 ± 0.09	1.02 ± 0.03

Note: Abbreviations are defined as follows: TC refers to total cholesterol; TG refers to triglyceride; TP refers to total protein; ALB refers to albumin; HDL-C refers to high-density lipoprotein; and LDL-C refers to low-density lipoprotein. Values with different lowercase letters in the same row differ significantly (*p* < 0.05).

**Table 4:** Effects of GML on immune and antioxidant parameters of *M. albus*.

Index	CON	GML0.5	GML1
ACP (mmol/min/g)	3.61 ± 0.14 ^a^	3.11 ± 0.15 ^b^	3.46 ± 0.18 ^a^
AKP (mmol/min/g)	1.80 ± 0.08 ^a^	1.42 ± 0.78 ^b^	0.43 ± 0.1 ^c^
SOD (U/g)	393.88 ± 56.65	391.88 ± 38.57	313.41 ± 77.40
MDA (nmol/g)	9.96 ± 0.97 ^a^	7.49 ± 0.84 ^b^	6.16 ± 0.02 ^b^

Note: Abbreviations are defined as follows: ACP stands for acid phosphatase, AKP for alkaline phosphatase, SOD for superoxide dismutase, and MDA for malondialdehyde. Values with different lowercase letters in the same row differ significantly (*p* < 0.05).

**Table 5:** Effects of GML on intestinal histomorphology of *M. albus*.

Index	CON	GML0.5	GML1
VH (μm)	814.91 ± 25.23 ^c^	1035.02 ± 83.07 ^b^	1175.08 ± 48.42 ^a^
VW (μm)	465.47 ± 36.70 ^c^	677.90 ± 52.90 ^b^	759.25 ± 45.97 ^a^
MT (μm)	155.05 ± 28.86 ^c^	178.33 ± 12.22 ^b^	202.39 ± 5.94 ^a^

Note: Abbreviations are defined as follows: VH refers to intestinal villus height; VW refers to intestinal villus width; and MT refers to intestinal muscular thickness. Values with different lowercase letters in the same row differ significantly (*p* < 0.05).

**Table 6:** Adonis analysis of intestinal microbiota β-diversity.

	Df	SumOfSqs	R^2^	F	Pr (>F)
Group	1	0.48	0.20	2.49	0.01
Residual	10	1.94	0.80		
Total	11	2.43	1		

The abbreviations in the table are defined as follows: Df refers to Degrees of Freedom, which reflects the number of independent pieces of information in the data; SumOfSqs stands for Sum of Squares, a parameter used to measure the degree of data variation; R^2^ denotes the Coefficient of Determination, representing the proportion of total variation explained by grouping factors (ranging from 0 to 1); F is the F statistic, which measures the ratio of between-group variation to within-group variation; Pr (>F) is the *p*-value, i.e., the probability of observing the current or a larger F statistic; Group indicates the effect of grouping factors; Residual refers to residual variation (within-group variation), namely the random error that cannot be explained by grouping; and Total represents total variation, which is equal to the sum of squares of Group and Residual.

## Data Availability

The original contributions presented in the study are included in the article; further inquiries can be directed to the corresponding author.
